# Metabolic syndrome and its components among university students in Kenya

**DOI:** 10.1186/s12889-017-4936-x

**Published:** 2017-11-28

**Authors:** Samuel Mungai Mbugua, Samuel Thuo Kimani, Gilbert Munyoki

**Affiliations:** 1 Mount Kenya University, School of Nursing, Thika, 342-01000 Kenya; 2 University of Nairobi, College of Health Sciences, School of Nursing Sciences, P.O Box 19676, KNH, Nairobi, Kenya; 3 Kenyatta University, School of Medicine, P.O Box 43844, Nairobi, 00100 Kenya

**Keywords:** Metabolic syndrome, University students, Diabetes

## Abstract

**Background:**

Metabolic syndrome refers to a cluster of interrelated disorders which occur together causing an increase in the risk of developing cardiovascular disease and diabetes. The university population is an understudied group despite the increase in the frequency of related disorders and metabolic risk factors e.g. obesity and diabetes, majorly due to the assumption that they are in their most active phase of life therefore healthy. This study looked at metabolic syndrome, the sedentary lifestyles and dietary habits present among university students attending Mount Kenya University, main campus.

**Methods:**

Stratified sampling was used to select participants. Self-administered questionnaires were issued to participants after a signed consent had been obtained following which clinical assessments and biochemical measures were performed. They included blood pressure, fasting blood glucose, triglycerides, high density lipoprotein-cholesterol, anthropometric measurements; height, weight, BMI and waist circumference. Pearson’s chi-square tests and non-parametric independent t-test were used to analyze the prevalence of metabolic syndrome criteria per gender, the number of metabolic syndrome criteria per BMI and prevalence of metabolic syndrome criteria per BMI category.

**Results:**

The study established that 1.9% of the participants met the criteria for diagnosis of metabolic syndrome according to HJSS criteria. Among the elements, there was statistical difference in gender BMI and waist circumference. 11.8% of subjects had two metabolic syndrome components while 3.1% had three components while none of the subjects had all six components. Elevated triglycerides was the most prevalent defining component for metabolic syndrome. There is a statistically significant relationship between sedentary lifestyle and dietary habits as risk factors to metabolic syndrome.

**Conclusion:**

Young adults in university have begun developing metabolic syndrome and the risk of developing the syndrome continues to increase with the components being reported in early age. Educational initiatives to encourage healthy eating should be conducted within school premises in order to reinforce the message on healthy diets and physical exercise. Pre-admission screening to identify at risk students should be conducted. Targeted interventions development through a mandatory extra co-curricular program should be enforced to positively engage those at risk.

## Background

Metabolic syndrome (MS) refers to a combination of disorders which when they occur together increase the risk of developing cardiovascular disease and diabetes. It is a cluster of interrelated cardio-metabolic risk factors that include insulin resistance, lipid imbalance and hypertension [1–4]. Metabolic syndrome puts people at a much higher risk for heart attacks, strokes, complications of diabetes, and sudden cardiac death [5].

MS has become a public health concern currently and in future as a result of a rapid increase in childhood-teenage obesity and sedentary lifestyles putting young adults at risk [6]. A depiction of this in the developed world was reported in the 2003–2006 National Health and Nutrition Examination Survey (NHANES) in the US population that indicated a prevalence of metabolic syndrome in males and females as 20.3% and 15.5% respectively aged between 20 and 39 years [7]. Obesity among college students aged 18–29 years has increased significantly globally [8–10]. Studies have shown that the propensity of having one of the defining criteria for metabolic syndrome in college students in the United States was between 26% and 40% [11]. The prevalence of metabolic syndrome in children and adolescents in comparison to adults is relatively low, however overweight and obese adolescents have a prevalence of metabolic syndrome of up to 29% [12, 13]. Dysmetabolic syndrome in childhood and adolescence has also been proven to increase the risk of developing metabolic syndrome and cardiovascular disease [14].

Early detection of metabolic syndrome is critical for commencement of directed interventions leading to reduction in the risk of progression to metabolic syndrome, coronary heart disease and diabetes [15, 16]. The risk factors for MS include central obesity, sedentary lifestyle, an elevated Body Mass Index (BMI), lack of physical exercise and poor dietary habits. Each of these factors has been shown to cause increased predisposition towards metabolic syndrome when they occur at an early age [11, 12].

Studies are required to understand the magnitude of the prevalence of MS among university students and despite the significant predisposition for obesity and other metabolic risk factors among university students, they remained a scarcely studied group and no study had been conducted in Kenyan universities.

Young people, aged 10–24 years constitute 36% of the total population in Kenya forming a population of about 25.8 million [17]. Early prevention of preceding risk conditions and emphasis on change in lifestyle eating behaviors and attention to exercise would reduce progression to metabolic syndrome and other cardiovascular conditions later in life.

## Methods

### Study population

Participants in this cross-sectional study were recruited from Mount Kenya University, main campus, located in Thika town on the outskirts of Nairobi, Kenya. The students attended university in Mount Kenya University in the main campus from all schools and were between the ages of 18–25 years. They were required to have enrolled in the regular mode of study with no previous chronic illness. Stratified sampling was employed with the stratification according to schools in the main campus. Participants were recruited through classroom announcements and word-of-mouth. 323 students were sampled to take part in the study. Signed consent was requested before taking part in the study. The questionnaires were number coded with each respondent having their own number assigned to them so as to ensure anonymity. Biochemical measures were collected and analysis performed with confidentiality assured.

### Lifestyle and health data

Data on socio-demographics, sedentary lifestyle and dietary habits was collected using self-administered questionnaires at the Nursing Skills laboratory on arrival for screening following a 12-h fasting exercise.

After a five minutes rest, a self-administered questionnaire earlier developed and validated was distributed to consenting participants and once they finished filling them, biochemical measures, anthropometric measurements of Body Mass Index and waist circumference were conducted. The biochemical measures and anthropometric measurements were performed by a trained nurse.

Weight, height, waist and hip circumference were performed by qualified nurse. Height was taken using a Seca Rod 220 stadiometer (Seca, Hamburg, Germany), weight using a TANITA weighing scale (TANITA, Arlington Heights, Illinois) and waist circumference using a Gulick tape measure. The participants were required to wear light clothing, no shoes and with or without socks during weighing. BMI was calculated using the formula: weight in kilograms/height in meters squared (kg/m^2^) and the WHO guidelines on body mass index were utilized. Waist circumference was measured at the top of the iliac crest upon exhalation. Systolic and diastolic blood pressure was taken flowing a five minutes rest on arrival using an automated monitor. The participating students were given a copy of their individual results and the health take in the findings.

### Biochemical measures

Following a 12-h minimum fast, fasting blood samples were assayed by standard methods for glucose, HDL-cholesterol and triglycerides. The blood samples were analyzed using a SD Cholesterol/Lipid desktop biosensor analyzer. The point-of-care SD Biosensor analyzer gives a lipid profile and glucose levels in three minutes. Participants were each given a copy of their results and for those whose values were outside normal parameters, they were referred for follow up with their healthcare provider.

### Metabolic syndrome diagnosis criteria

The following criteria stipulated by the Harmonized Joint Scientific Statement (HJSS) on metabolic syndrome were used [18]. Blood pressure- > 130/85 mmHg. (Hypertension), Impaired glucose handling-PG >200 mg/dL (11.1 mmol/L), raised fasting plasma glucose >100 mg/dL (>5.6 mmol/L), Waist circumference: >94 cm (male) >80 cm (female), Body mass index >30 kg/m^2^, Dyslipidemia: triglycerides (TG): ≥ 1.7 mmol/L /150 mg/dL and high-density lipoprotein cholesterol (HDL-C) ≤ 1.0 mmol/L/40 mg/dL (male), ≤ 1.3 mmol/L /50 mg/dL (female). To make a diagnosis of metabolic syndrome, the study considered any three characteristic abnormalities.

### Statistical analysis

Statistical Package for Social Sciences (SPSS) version 22.0 was used for analysis. Demographics were calculated using means and frequencies. Analysis of variance (ANOVA) scores on the means procedure were used to examine differences between all anthropometric, clinical and biochemical parameters. Pearson’s chi-square tests and non-parametric independent t-test were used to analyze the point prevalence of metabolic syndrome criteria per gender, the number of metabolic syndrome criteria per BMI and prevalence of metabolic syndrome criteria per BMI category. The *p*-value was set at 0.05 to test for significance in all tests.

## Results

Demographics on the participants, individual defining criteria of metabolic syndrome, anthropometric, clinical and biochemical parameters in males and females are shown in Table 1.

**Table 1:** Anthropometric, clinical, and biochemical description of the subjects^a^

Characteristics	Gender	N	Mean	Std. deviation
Age (Years)	Male	116	23.2	4.0
	Female	207	21.8	2.7
BMI (Kg/m^2^)	Male	116	21.7	3.1
	Female	207	23.3	3.9
Waist circumference (Cm)	Male	116	77.7	7.2
	Female	207	77.4	9.4
Systolic Blood Pressure (mmHg)	Male	116	128.3	12.5
	Female	207	116.8	12.5
Diastolic Blood Pressure (mmHg)	Male	116	75.1	9.5
	Female	207	74.1	8.4
Fasting blood glucose (mmd/l)	Male	116	4.8	0.6
	Female	207	5.1	3.2
Triglycerides (mg/dl)	Male	116	122.6	67.7
	Female	207	115.9	72.1
High Density Lipoprotein (HDL) cholesterol (mg/dl)	Male	116	58.1	20.7
	Female	207	59.5	18.1
Total cholesterol (mg/dl)	Male	116	152.6	38.1
	Female	207	171.4	38.5
Low Density Lipoprotein (mg/dl)	Male	116	71.3	32.6
	Female	207	76.8	39.1
Non - High Density lipoprotein (mg/dl)	Male	116	114.8	37.5
	Female	207	102.4	41.2

^a^Analysis of Variance (Anova)

Out of the 323 students participating in the study, majority 64.1% (*n* = 207) were female and 35.9% (*n* = 116) were male. The mean age for males was 23.2 years (SD = 4.0) while the mean for females was 21.8 (SD = 2.7) (Table 2).

**Table 2:** Represents the independent T-test results for equality of means of the defining criteria^a^

	Levene’s test for Equality of Variances		t-test for Equality of Means		
	F	Sig.	t	Df	Sig. (2-tailed)
Age of the respondent	3.591	.059	3.848	321	.001
Calculated BMI (Kg per square metre)	4.209	.041	4.330	236.964	.001
Waist circumference of the respondent in centimeters	9.535	.002	.270	291.303	.787
Systolic Blood Pressure (mmHg)	.341	.560	7.919	321	.001
Diastolic Blood Pressure (mmHg)	1.566	.212	.974	321	.331
Triglycerides (mg/dl)	.627	.429	.820	321	.413
High Density Lipoprotein (HDL) cholesterol	.779	.378	−.426	321	.670
Total cholesterol (mg/dl)	.264	.608	−4.228	321	.001
Low Density Lipoprotein (mg/dl)	2.544	.112	−1.290	321	.198
Non - High Density lipoprotein (mg/dl)	3.327	.069	1.207	321	.228

^a^Independent t-test

It was established that elevated triglycerides was the most prevalent defining criteria for metabolic syndrome with 24.8% of the subjects diagnosed with the symptom. Low high density lipoprotein cholesterol was the second most prevalent defining criteria for metabolic syndrome, followed by impaired fasting glucose and high blood pressure respectively. 69.3% of the subjects were in the normal range; however 3.7% were obese and 19.5% were overweight (Table 1).

**Fig. 1:**
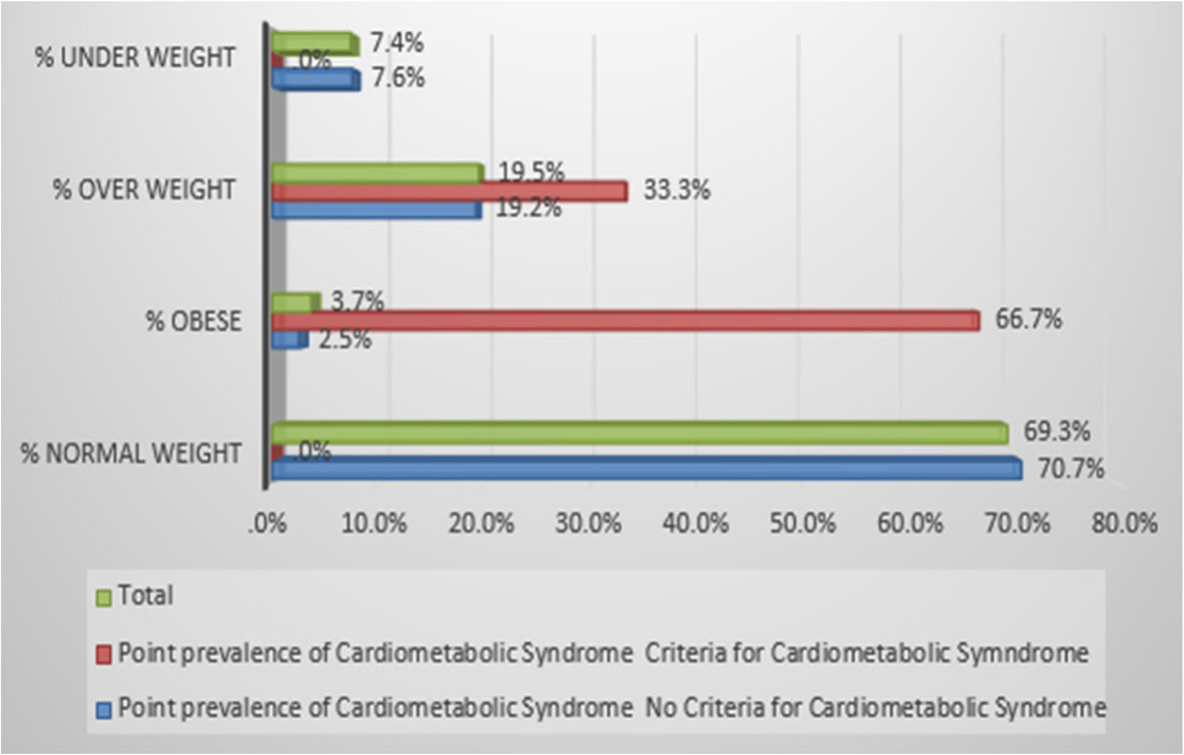
BMI Category and point prevalence of Metabolic syndrome

The study established that 1.9% of the subjects met the Harmonized Joint Scientific Statement on Metabolic syndrome criteria for diagnosis of metabolic syndrome with a confidence interval (CI) of 95%. In regards to gender differences, it was established that out of the six subjects diagnosed with metabolic syndrome, five were females while only one was a male. Of the six subjects that were diagnosed with metabolic syndrome, four were obese, two were overweight and none was in either the normal weight category or underweight category (Fig. 1). The relationship between BMI and metabolic syndrome was statistically significant, 1(*p* < .001).

**Fig. 2:**
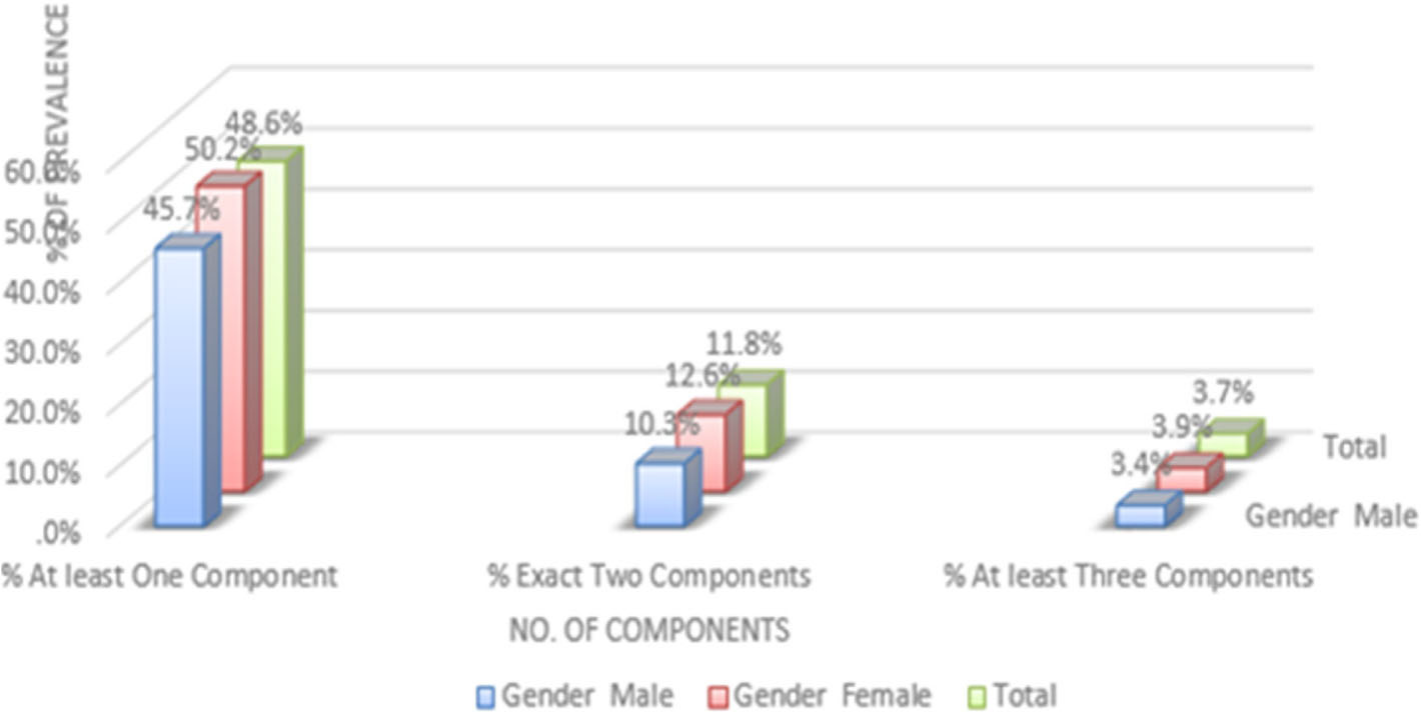
Gender differences in regard to prevalence of metabolic syndrome components

It was established that 50.2% of the female subjects recorded at least one component of metabolic syndrome which was slightly higher than that of their male counterparts at 45.7% (Fig. 2).

**Fig. 3:**
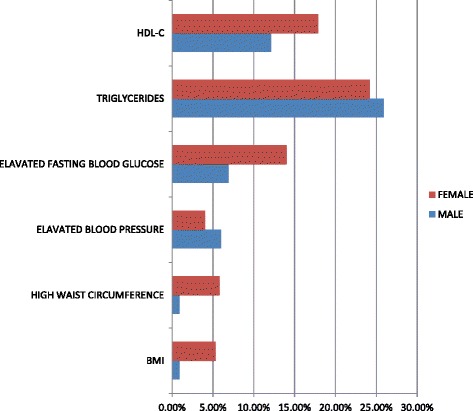
Gender differences in regards to elevated metabolic syndrome elements

5.8% of the females’ subjects had a high waist circumference compared to 0.9% of their male counterparts. The percentage of females that had a high waist circumference was six times as much as the percentage of males (Fig. 3). The difference in waist circumference between the genders was statistically significant (*p* < 0.05).

77.6% of the males were within the normal weight, 12.1% were overweight, 9.5% were underweight and 0.9% were obese. 69.3% of females were within the normal weight range, 19.5% were overweight, 7.4% were underweight and 3.7% were obese. The relationship between BMI and gender was statistically significant (*p =* 0.008).

**Fig. 4:**
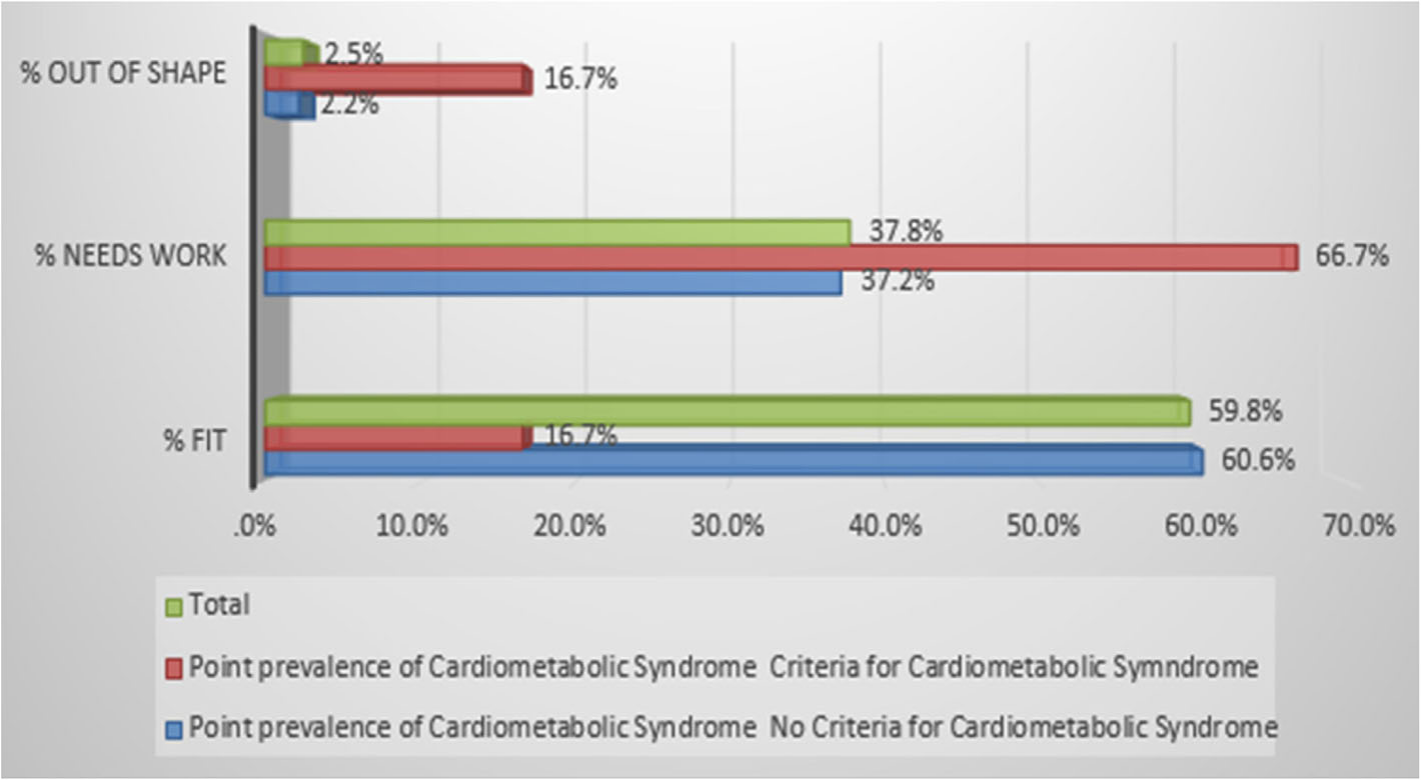
Sedentary lifestyle and point prevalence of Metabolic syndrome

In order to assess sedentary lifestyle physical exercise, sleep duration, snacking habits and normal daily activity were considered in the self-administered questionnaire. 61.3% of the respondents reported that they did not exercise frequently (Fig. 4). The average sleep duration for 52.9% of respondents was 7 to 8 h with 31.3% reporting 5 to 6 h. 52.3% indicated limited activity in their day and 72.8% snacked on various processed foods while watching television or on their computers. The relationship between sedentary lifestyle as a risk factor and metabolic syndrome was statistically significant, (χ2 = 8.221, df = 2, *p* = 0.016).

**Fig. 5:**
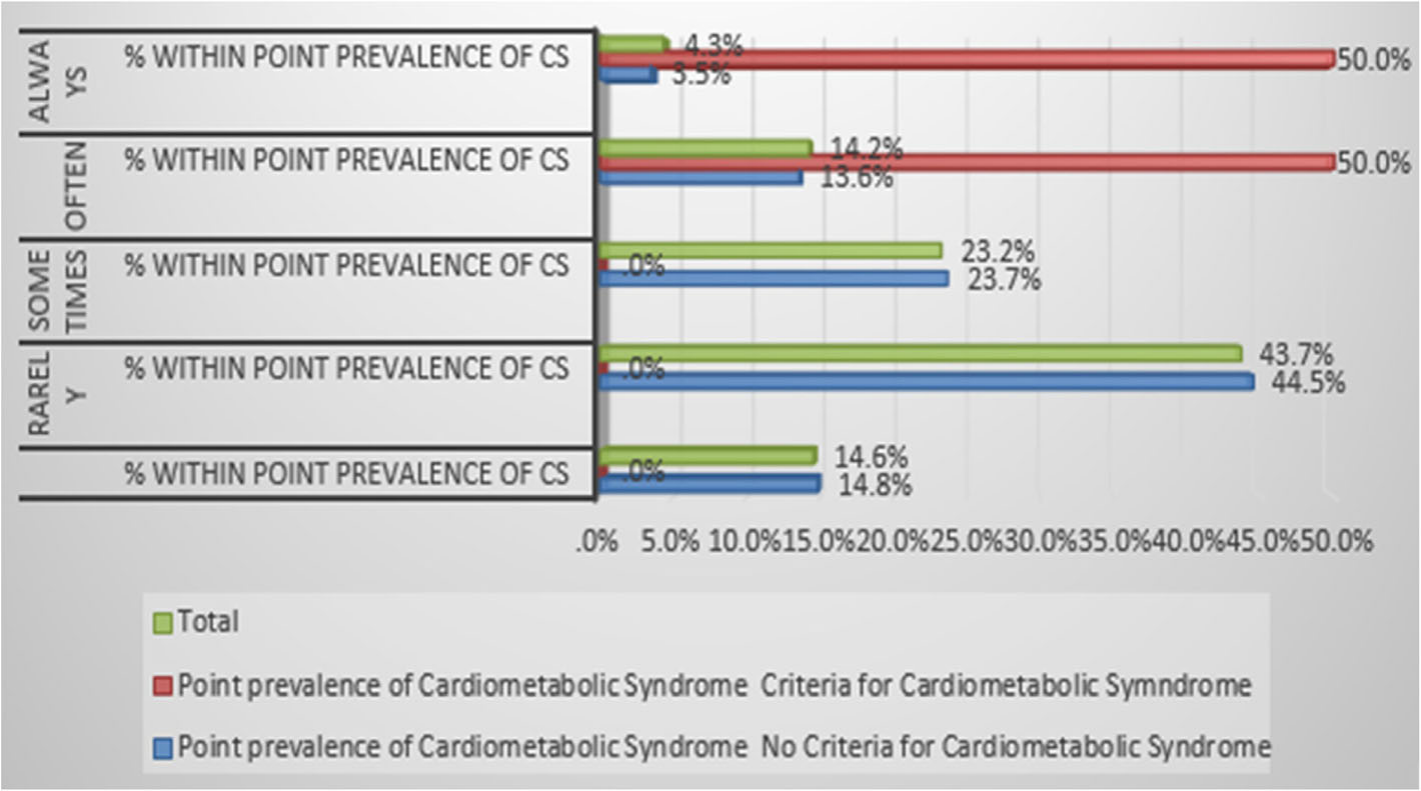
Dietary Habits and Point prevalence of Metabolic Syndrome

Data on diet and dietary habits showed that only 13.3% of the participants reported good nutritional intake while the remaining 84.7% were concerned about their nutritional status. 9.3% of respondents had poor eating habits. 85.4% of participants were not aware of the amount of calories consumed in a day. 32.8% of respondents reported to take at least one soft drink in a day (Fig. 5). The relationship between dietary habits as a risk factor and metabolic syndrome was statistically significant, (χ2 = 39.881, df = 4, *p* < 0.001).

## Discussions

There is a general misconception that young adults in ages between 18 and 25 years are healthy but despite this metabolic syndrome is present with 1.9% of the sample having MS. The most prevalent defining criteria was elevated triglycerides (24.8%) followed by low high density lipoprotein cholesterol (15.8%) with high waist circumference being the least prevalent. This is similar to studies by Huang et al., Dalleck & Kjelland and Fernandes & Lofgren [11, 12, 15]. Significantly more females were found to meet MS diagnosis criteria compared to their male counterparts. Though there is an impression that university students are healthy, this study reveals an occurrence of MS in these young adults and necessity for extensive health screening, education and sensitization to reduce the progression towards MS.

The prevalence of MS identified in this sample population correlates with similar studies of Huang et al. (1.7%) and Fernandes & Lofgren (3.7%), [11, 15]. It was lower than the findings by Tope & Rogers (9.3%) and Yen et al. (4.6%) [8, 19]. This can be attributed to differences in lifestyle choices and dietary habits as most of the studies were carried out in the USA and none in Eastern Africa. Morell et al. reported higher metabolic syndrome prevalence in males than in the females [20]. This could be speculated as a result of differences in socio-economic backgrounds and race. 32.8% of the subjects had one component of metabolic syndrome, 11.8% had two components and 3.1% had three components. This is similar to studies by Tope & Rogers and Fernandes & Lofgren [8, 15]. This emphasizes the need for health screening of university students as additional defining criteria could develop later in life resulting from sedentary lifestyle and poor dietary habits.

The relationship between gender and waist circumference is statistically significant with more females having a high waist circumference compared to the males; similarly, there is statistical significance between systolic BP and gender but no significance between diastolic BP and gender. Although the percentage of female subjects with impaired fasting glucose was twice that of their male counterparts, similar to the findings by Huang et al., there is no statistical significance between gender and fasting blood glucose [11]. The study established that there is a statistically significant relationship between BMI and metabolic syndrome. Similarly, there is a statistically significant relationship between gender and body mass index with the number of females having an elevated BMI being four times more that of the males. This is similar to Tope & Rogers [8]. It however differs from the findings by Huang et al. where the males were found to be more obese than the females [11]. This could be attributed to the demographical and socio-economical differences between the study populations. Gender differences in regard to overall prevalence of metabolic syndrome was established indicating a higher prevalence in females since out of the six subjects diagnosed with metabolic syndrome, five were females. Significance could not be tested as only one male met the MS criteria. This correlates to findings by Tope & Rogers and Fernandes & Lofgren [8, 13]. The importance of screening and early intervention is critical in early management and prevention of MS in university students. This is partly because most of the sedentary lifestyle patterns and dietary habits in this stage of life persist later in adulthood increasing the likelihood of progression to MS indicating the significance of health screening in a university population.

## Conclusion

This being one of the few studies on MS in university students in Africa and the first in Kenya, the prevalence of MS and each of the defining criteria is significantly high considering the predisposing risk to MS and chronic heart disease in later life. Educational initiatives on the importance of regular physical exercise and dietary modifications in terms of choices and habits are key in improving the cholesterol, triglyceride, glucose and HDL-C in the university population. Health screening measures are vital in the identification of at-risk young adults and the implementation of targeted interventional development. There is need for further research in this population to aid in development of a through screening and intervention policy for university students.

## Abbreviations

ANOVA: Analysis of Variance
BMI: Body Mass Index
BP: Blood pressure
CI: Confidence Interval
HDL-C: High density lipoprotein cholesterol
MS: Metabolic syndrome
SPSS: Statistical Package for Social Sciences

## Definition of Terms

**Young Adults**: In this study, young adults are defined as persons aged 18–25 years.
**Sedentary lifestyle**: A lifestyle characterized by little physical exercise, frequent/extended sleep, long durations of sitting, watching television, playing video games, using a computer/mobile phone, and is usually accompanied by snacking habits.
**Dietary habits**: Decisions made by an individual regarding choice of food, preferences, content (diet), calorie intake and frequency of consumption.

## Footnotes

1 Chi-square test
